# Trachoma and the Need for a Coordinated Community-Wide Response: A Case-Based Study

**DOI:** 10.1371/journal.pmed.0030041

**Published:** 2006-02-28

**Authors:** Heathcote R Wright, Jill E Keeffe, Hugh R Taylor

## Abstract

Wright and colleagues discuss the diagnosis and management of trachoma, both at the individual and community level.

## DESCRIPTION of CASE

A young nurse, undertaking a working holiday in Australia, sees a 5-year-old Aboriginal girl while doing a locum in a remote coastal community of the Northern Territory. The young girl has been brought in by her grandmother because she has discharge coming from her eyes. From the notes, the nurse sees that the child has had multiple presentations for ear and skin infections but is otherwise well. The child's immunisations are up to date.

The girl denies having any pain but otherwise cannot provide much history; neither can her grandmother, who states that the girl has been staying with an aunt. Upon examination there is a small amount of mucopurulent discharge, her visual acuity is normal, and the rest of the examination is unremarkable. A diagnosis of bacterial conjunctivitis is made, and the nurse is about to send the young girl home with some antibiotic eyedrops, when she decides to mention the girl's problem to the senior clinic nurse.

### Are the Diagnosis and Treatment Adequate?

The senior nurse agrees that a mucopurulent discharge suggests a mild bacterial infection, but is concerned about the possibility of a chlamydial infection. Trachoma is known to be hyperendemic in Central Australia; however, the prevalence in coastal communities is not well documented. Trachoma thrives in dry, dusty regions and is less common in tropical or coastal areas; however, the disease can thrive anywhere that overcrowding and poor hygiene is prevalent. When they examine the patient's everted eyelids with 2.5× loupes and the aid of good illumination, they find follicles clearly visible on the underside of the upper eyelid.

### What Is Trachoma?

**Figure 1:**
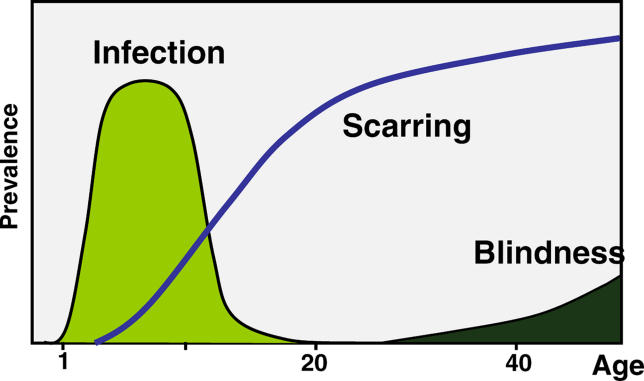
Natural History of Trachoma Trachoma has a natural history consisting of three stages: active disease with inflammatory follicles, scarring, and trachomatous blindness.

**Figure 2:**
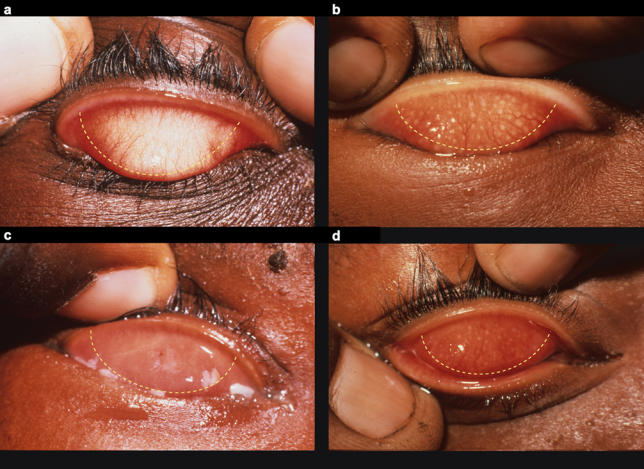
Active Disease Is Characterised by Inflammatory Follicles Signs are considered only if they appear within the area denoted by the dotted line. (A) Normal conjunctiva. (B) TF. (C) TI. (D) TF and TI.

Trachoma is a keratoconjunctivitis caused by the bacterium Chlamydia trachomatis. It is a disease with distinct stages ( Figure 1). These stages generally correspond to the five signs that are assessed by the World Health Organization (WHO) simplified grading system ( Table 1); however, the stages are not mutually exclusive and more than one and sometimes all can coexist in the same eye [ 1]. Active inflammation results from repeated or persistent infection, predominantly in children, and is characterised by the presence of follicles on the tarsal conjunctiva (undersurface of the eyelid). Such active inflammation is graded as trachomatous inflammation follicular (TF) and/or trachomatous inflammation intense (TI) ( Figure 2).

**Table 1:**
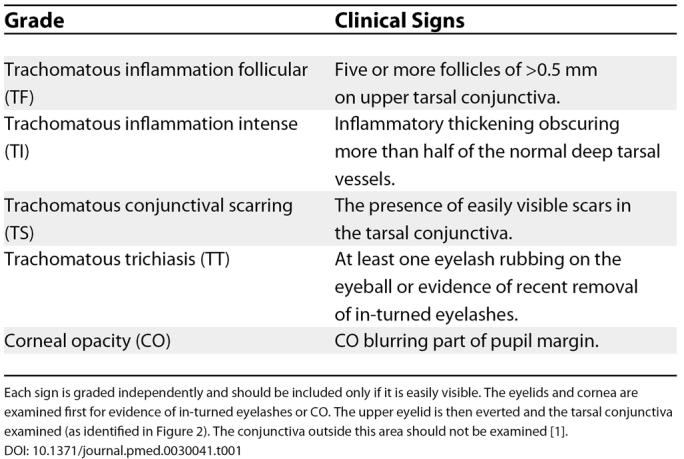
Simplified WHO Grading System

**Figure 3:**
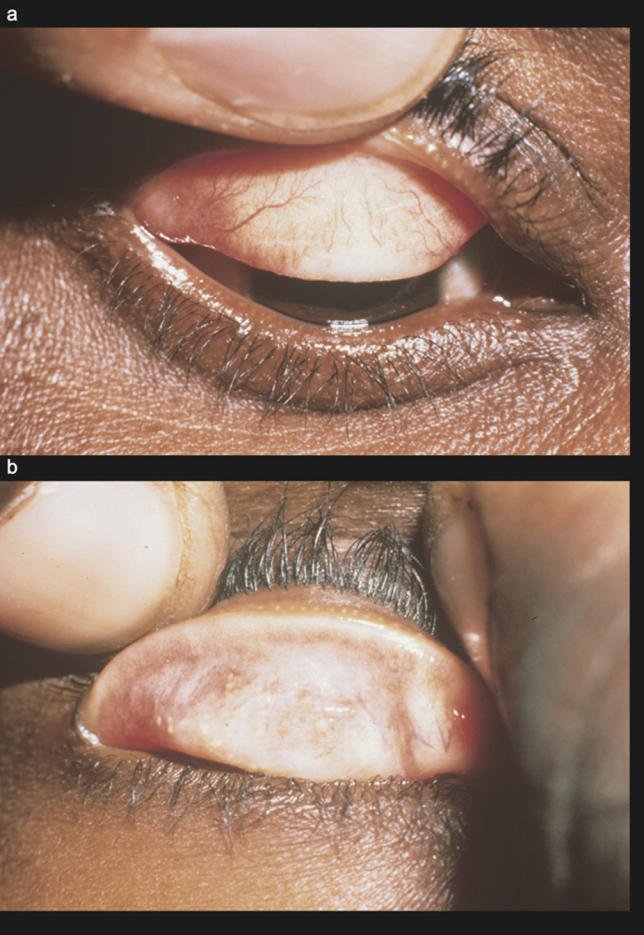
TS Develops in Response to the Inflammation of Active Disease (A) TS: fine transverse lines crossing over the vessels. (B) Severe scarring.

**Figure 4:**
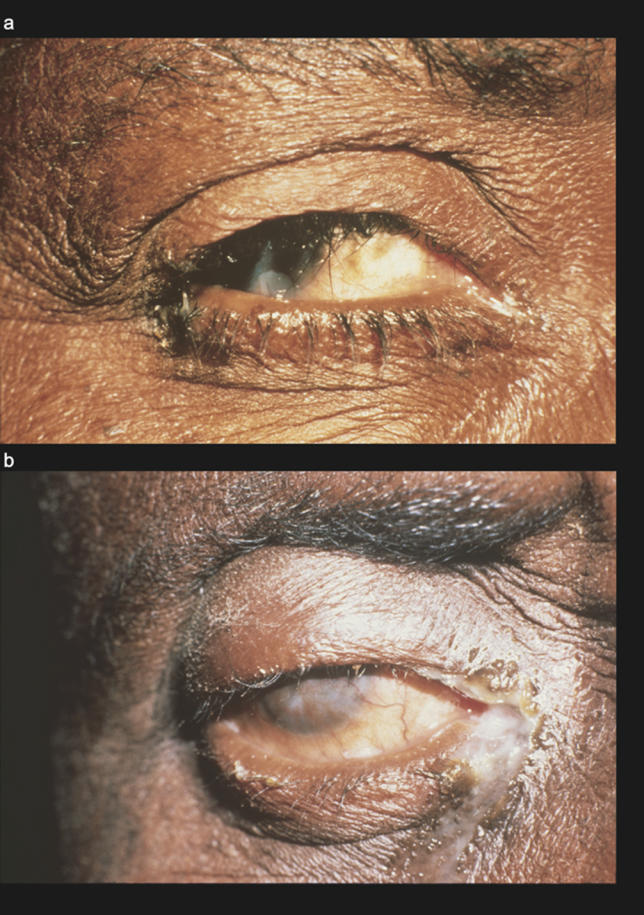
Cicatricial Disease (A) TT scarring and contraction has pulled the lid margin in, and eyelashes now touch the globe. (B) CO results from the constant abrasion of lashes on the cornea.

Cicatricial disease is the end result of scarring that occurs in individuals who suffer from severe, persistent, or frequent infections in childhood. Trachomatous scarring (TS) is recognised by the presence of easily visible white bands on the underside of the upper eyelid, and is more prevalent with increasing age ( Figure 3). Severe scarring can result in contraction of the tarsal conjunctiva; this causes the lid margin to roll inwards and results in lashes contacting the globe. Called trachomatous trichiasis (TT), this is more common with increasing age; however, the age of onset varies according to the severity of disease within the community. The constant abrasions of the lashes on the cornea are painful and rapidly produce scarring and corneal opacities (CO) ( Figure 4). Once the cornea has become opaque, visual loss is essentially irreversible.

## How Do You Diagnose Trachoma?

Trachoma is generally diagnosed as a part of a screening program, and will not be diagnosed unless specifically looked for. It is generally asymptomatic, and only a minority of cases will have a mucopurulent discharge.

In a patient presenting with a mucopurulent discharge, one should consider trachoma or another bacterial infection. The presence of follicles in the case of this Aboriginal girl would support the diagnosis of trachoma. However, most patients with follicles will be asymptomatic. ( Table 2 provides a differential diagnosis for follicular conjunctivitis.)

**Table 2:**
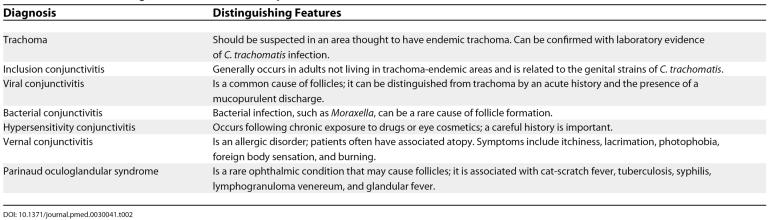
Differential Diagnoses for Follicular Conjunctivitis

If available, polymerase chain reaction (PCR) can be used to distinguish between these different conditions in the differential diagnosis (direct fluorescence antibody [DFA] techniques, if available, can be substituted for PCR). However, these laboratory techniques are generally not of much practical benefit. Not all people with active trachoma (TF and TI) will be positive for C. trachomatis, and both tests (PCR and DFA) will be positive in paratrachoma (a disease caused by the genital strains of C. trachomatis). Therefore, the diagnosis of trachoma in an individual should be made on the basis of clinical signs and epidemiology of the disease within the community. The presence of conjunctival follicles in an area known to have hyperendemic levels of trachoma is highly suggestive of C. trachomatis infection.

## How Was the Patient Treated?

A swab of the young girl's eyelid was taken and sent to Darwin (capital of the Northern Territory). PCR analysis confirmed the diagnosis of trachoma. The girl was given presumptive treatment, at the initial consultation, with a single dose of oral azithromycin (20mg/kg), and reviewed a week later. At review the discharge had cleared up, but the follicles remained. Follicles are thought to remain for up to three months or so, even after infection has been cleared.

## What Was the Public-Health Response?

The Centre for Disease Control in Darwin was notified, and an action plan was implemented. An ophthalmologist was flown into the community, and all children aged 1–10 years were screened. Each child was examined in the shade; their upper eyelids were everted and inspected with the aid of 2.5× loupes and a good light source. The presence of TF and/or TI was recorded. The prevalence of trachoma (TF or TI) was 35%, with 5% having TI.

The results of the survey were reported back to the Centre for Disease Control, and a decision was made to initiate a mass treatment campaign. The effective treatment of trachoma requires the implementation of the SAFE (surgery, antibiotics, facial cleanliness, and environmental improvements) strategy. A repeat survey was conducted 12 months later, and the prevalence of trachoma was considerably decreased.

## DISCUSSION

Trachoma is the leading cause of infectious blindness worldwide; it is endemic in 48 countries throughout the world and may affect as many as 84 million people [ 2]. Treating individuals with trachoma in isolation is futile because the disease is characterised by easy transmission and repeat infections. Therefore, a treated individual will be rapidly re-infected by those who have not been treated. At a bare minimum all household contacts must be treated, and ideally all components of the SAFE strategy should be implemented on a community-wide basis. The first step in doing this is to estimate the prevalence of trachoma.

### How Can You Estimate the Prevalence of Trachoma?

Instigation of a trachoma treatment program should follow guidelines provided by the WHO. Such a program will depend on the prevalence of active disease in children 1–10 years old, the availability of resources, and the acceptance the program will have in the community. In larger communities or where resources are scarce, it may be impractical to examine all children. In such cases, one of a number of survey techniques can be used to estimate the prevalence of trachoma [ 3].

Population-based prevalence surveys are the gold standard for estimating the level of trachoma within a community, and the WHO has published guidelines detailing how these surveys can be conducted [ 4]. Prevalence surveys, however, are expensive, time consuming, and may use resources that could be better spent on intervention programs. Alternate tools have been developed that more efficiently estimate the burden of disease (these tools do not provide prevalence estimates).

#### Trachoma Rapid Assessment (TRA).

This tool, developed by the WHO [ 5], attempts to quickly, cheaply, and efficiently obtain the information needed to identify and prioritize areas for intervention programs. It uses a two-phase sampling technique to optimally bias the sample to detect people most likely to have trachoma. The first phase selects “at-risk” communities from a region on the basis of existing trachoma data and known socioeconomic conditions. This phase was not relevant in the above case of the Aboriginal girl, as the identification of an individual with trachoma immediately classifies the community as “at risk.” Once a community is considered to be “at risk,” 50 children from the “worst areas” of that community should be examined. The term “worst area” relates to those houses that can be identified as having risk factors for trachoma ( Table 3). This technique does not provide prevalence data, but what it does very well is exclude the presence of trachoma as a public-health problem. If the children living in houses with the greatest risk factors for trachoma do not have the disease, then it can be said with some degree of certainty that there is no trachoma within the community.

**Table 3:**
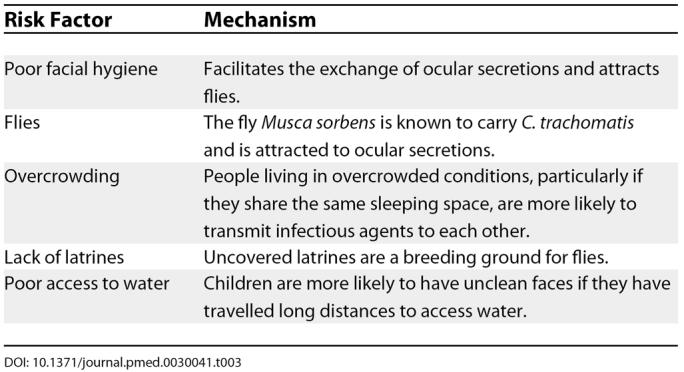
Risk Factors for Trachoma

#### Lot Quality Assurance Sampling (LQAS).

This is another alternative tool for the efficient assessment of trachoma. The method has long been used in manufacturing to monitor production quality [ 6]. When used as a tool for the rapid assessment of trachoma, the technique has been referred to as Acceptance Sampling Trachoma Rapid Assessment (ASTRA). Children are examined until either a predetermined number of cases with active disease is identified (high prevalence) or a total of 50 children are sampled without the cut-off point being reached (low prevalence). This method has the benefit of allowing the survey to stop as soon as it has sufficient information about the likely prevalence of trachoma to allow an intervention strategy to be planned.

### Should Clinical Examination or Laboratory Tests Be Used?

Clinical examination, in the form of the simplified WHO grading system, has proven to be easily learnt by local health workers and has generally shown a high level of reproducibility [ 7]. It is an essential tool in the effort to identify populations with trachoma and to monitor the progress of intervention programs. However, the need for laboratory-based confirmation of C. trachomatis infection, by the use of PCR testing, has been suggested. It is argued by some that clinical examination is unreliable, as it has only a 70% correlation with PCR in high-prevalence areas, and a 10% correlation with PCR in low-prevalence areas [ 8–10]. Moreover, after antibiotic treatment there is a more rapid decrease in PCR evidence of infection than there is an improvement of clinical signs [ 11, 12]. These findings have raised the concern that the use of clinical examination alone may result in the overtreatment of communities.

**Figure 5:**
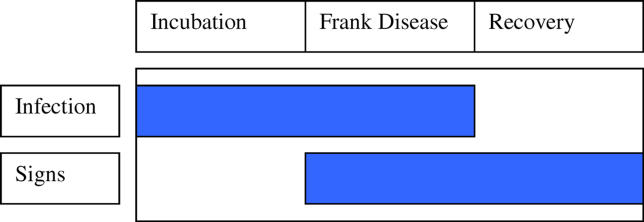
Diagrammatic Representation of the Three Distinct Stages of Active Trachoma: Incubation, Frank Disease, and a Possibly Lengthy Recovery Period

The lack of a strong correlation between clinical examination and PCR tests does not represent a deficiency in either test; rather, it reflects the complicated relationship between chlamydial infection and clinical signs of trachoma. Active inflammatory trachoma can be thought of as consisting of three distinct stages ( Figure 5). An initial incubation stage, during which infection can be identified but clinical signs are absent, is followed by frank disease, where clinical signs and infection coexist. The recovery phase may persist for a considerable period of time as clinical signs slowly resolve; however, during this time the infectious agent is not present. The duration of each stage is poorly understood; however, it is thought that the recovery stage may persist for three months or longer [ 13]. The picture is further complicated by repeat episodes of re-infection or persistent infection.

Episodes of chlamydial infection are a prerequisite for blinding trachoma; however, lack of demonstrable infection does not exclude trachoma. Current research is attempting to develop a cheap point-of-care PCR test. However, until such a product is available and its cost-effectiveness proven, clinical examination should remain the mainstay for assessing the need for, and monitoring the progress of, intervention programs [ 14].

### What Is the SAFE Strategy?

The SAFE strategy is being implemented on a global scale as part of the WHO initiative to eliminate trachoma as a cause of blindness by the year 2020. The strategy focuses on prevention; blindness from trachoma is essentially irreversible. Although international and national health bodies are generally responsible for the implementation of the SAFE strategy, it is community involvement and education about the disease at a local level that will result in elimination of trachoma as a blinding disease. The SAFE strategy has been shown to be an effective tool in reducing the blinding complications of trachoma and consists of four inter-related components [ 15].

Individuals with trichiasis have a greatly increased risk of becoming blind. These individuals need to be identified and operated on to reduce the pain of TT and to prevent or slow the progression toward blindness. The WHO recommends the bilamellar tarsal rotation procedure [ 16]. The finding of trachoma in children should prompt the active search for trichiasis in adults, particularly those over 40 years of age.

Antibiotics are the mainstay of treatment, and the two recommended antibiotics are azithromycin (single dose of 20 mg/kg) and tetracycline eye ointment (six-week course). Both have been shown to be effective in eliminating chlamydial infection and decreasing the prevalence of clinical signs [ 17]. Azithromycin is expensive; however, a donation program set up by Pfizer provides the drug free of charge to many countries in which trachoma is endemic. If the prevalence of trachoma is greater than 10% in children 1–10 years of age, then everyone in the community should be treated with antibiotics annually for at least three years [ 2]. Annual mass treatment should continue until the prevalence of trachoma is less than 5%. If available, azithromycin should be used, as compliance may be an issue with tetracycline.

Facial cleanliness needs to be promoted within the community, and adequate facilities for children to clean their faces need to be developed. Clean-face campaigns can significantly reduce trachoma, particularly intense trachoma. Children with dirty faces are two to three times more likely to have trachoma [ 18]. Environmental changes should attempt to address the risk factors for trachoma: overcrowding, poor access to adequate latrines, flies, and poor access to water.

### What Steps Should Be Taken to Monitor the Prevalence of Trachoma?

Once a treatment program has been instituted, it is important to monitor the effects of the program. A brief survey of selected areas should be conducted each year to ensure the performance of the program. If the prevalence of trachoma has risen again, then any problems in program implementation need to be developed and changes made. The institution of a trachoma intervention program will be different in every setting and should reflect the resources available to that area and must respect the cultural sensitivities of the population.

**Learning Points**

- Trachoma will not be seen unless it is specifically looked for, and if it is seen it should prompt the initiation of a community-wide public-health campaign.
- The magnitude of the problem, including the prevalence of active trachoma in children and trichiasis in adults, should be estimated.
- The individual components of the SAFE strategy should then be incorporated in a manner specific to the cultural, geopolitical, and financial constraints of the community.
- An ongoing process to monitor the prevalence of disease and the mechanics of the intervention program must then be set up.
- Without the institution of a coordinated community-wide public-health program, the treatment of individuals with trachoma is a futile gesture.

## Abbreviations

CO: corneal opacity
PCR: polymerase chain reaction
SAFE: surgery
TF: trachomatous inflammation follicular
TI: trachomatous inflammation intense
TS: trachomatous scarring
TT: trachomatous trichiasis
WHO: World Health Organization
